# Strawberry carina as a presentation of anti-neutrophil cytoplasm antibody–associated vasculitis

**DOI:** 10.1093/rheumatology/keab783

**Published:** 2021-10-20

**Authors:** Gavin B Chapman, Andrew E Leitch, Rashmi Lahiri, Peter Reid, Neeraj Dhaun

**Affiliations:** 1 British Heart Foundation Centre of Research Excellence, Centre for Cardiovascular Science, Queen’s Medical Research Institute, University of Edinburgh; 2 Department of Renal Medicine, Royal Infirmary of Edinburgh; 3 Department of Respiratory Medicine, Western General Hospital; 4 Department of Pathology, Royal Infirmary of Edinburgh, Edinburgh, UK

**Rheumatology key message**

- Strawberry-like inflammation of the airways may be a disease feature in patients with AAV.

Dear Editor, A 51-year-old man presented with a 7-week history of dry cough, shortness of breath and ankle swelling. There was no past medical history and the patient took no regular medications. He had never smoked and drank ∼20 units of alcohol per week. He had worked as a stonemason for 20 years.

**Fig. 1:**
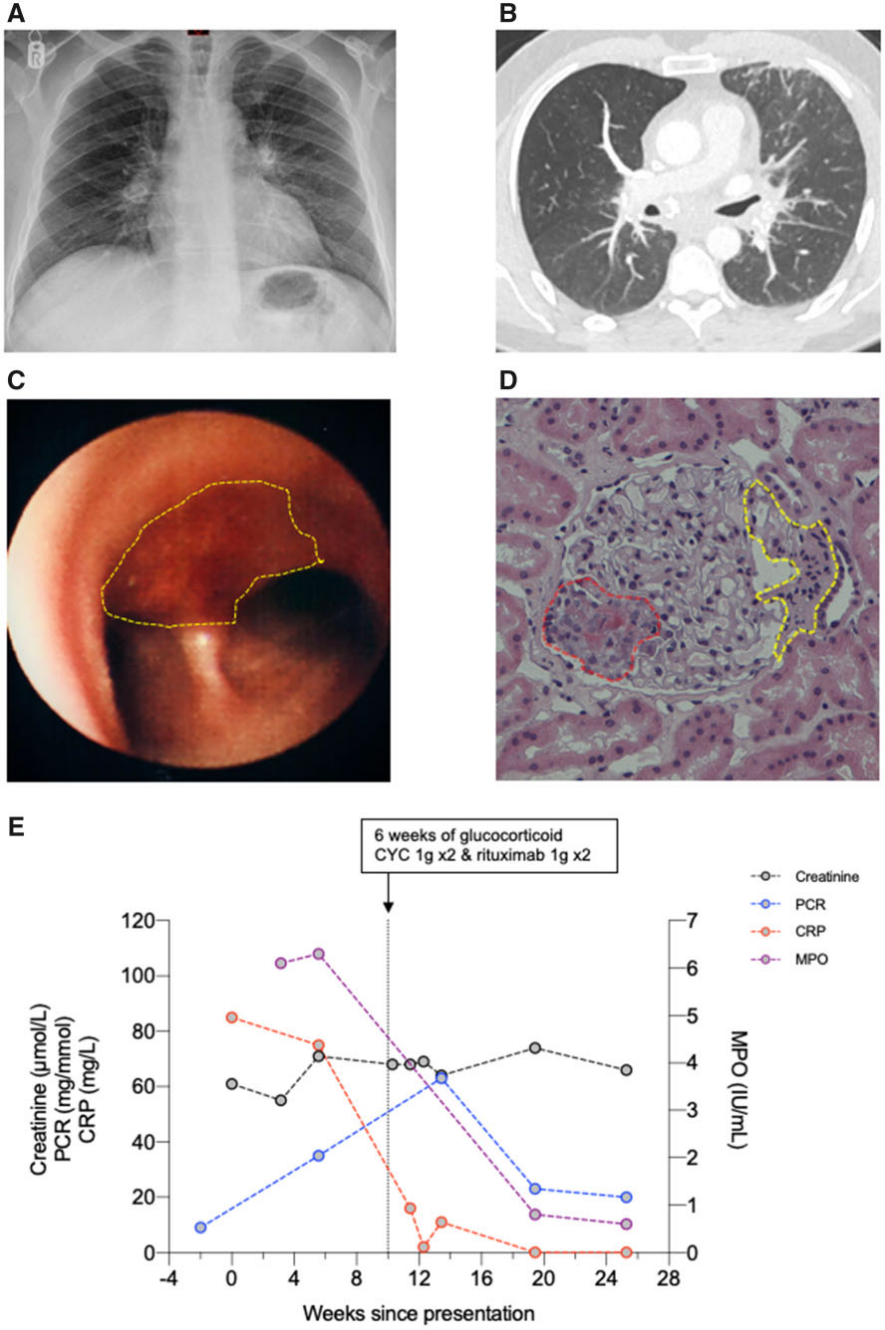
Radiological, pathological and biochemical results (**A**) Chest X-ray on admission showing enlarged, calcified hilar lymph nodes. (**B**) Axial view of CT of the chest demonstrating enlarged calcified hilar and subcarinal lymph nodes with minor left upper lobe interstitial and nodular change. (**C**) Bronchoscopic image demonstrating normal airway in foreground with discrete, haemorrhagic plaque (yellow circle) straddling the main carina with small, yellow pus islands (yellow line) reminiscent of a strawberry. (**D**) Kidney biopsy demonstrating glomerulus with necrotizing lesion (red line) and cellular crescent (yellow line) secondary to AAV. (**E**) Haematological and biochemical results from presentation. Vertical dashed line at 10 weeks representing when treatment commenced. PCR: protein:creatinine ratio.

On examination, there was bilateral ankle oedema but no other clinical findings of note. Blood tests showed a systemic inflammatory response with anaemia [115 g/l (normal range 130–180)], thrombocytosis [418 × 10^9^/l (normal range 150–400), elevated CRP [85 mg/l (normal range 0–5 mg/l)] and hypolbuminaemia [29 g/l (normal range 36–47)]. Kidney excretory function was normal [serum creatinine 61 µmol/l (normal range 64–111)] and there was no haematuria or proteinuria [urine protein:creatinine ratio 9 mg/mmol (normal range 0–30)]. A chest X-ray showed prominent hila bilaterally with some calcification (Fig. 1A). A CT scan of the chest, abdomen and pelvis with contrast demonstrated bilateral hilar and mediastinal lymphadenopathy with coarse calcification (Fig. 1B). There was bronchial wall thickening and ground-glass changes evident in the left upper lobe.

The patient was reviewed in the general respiratory clinic 10 days later. He had ongoing cough and breathlessness and now complained of right foot pain and drenching night sweats. The CT chest findings were in keeping with chronic silica exposure, although the differential included mycobacterial infection, sarcoidosis and lymphoma. Given the patient’s systemic symptoms, an endobronchial ultrasound bronchoscopy was performed to obtain a tissue diagnosis. On direct visualization, the airways were grossly abnormal, and the patient went on to have a flexible bronchoscopy that showed discrete haemorrhagic plaque straddling the main carina with small, yellow pus islands (reminiscent of a strawberry; Fig. 1C). There were further similarly discrete areas of haemorrhagic plaque in the left and right main bronchus. The intervening bronchial mucosa was normal. Bronchoalveolar lavage showed a minor excess of inflammatory cells. Biopsies of the bronchial wall showed only a minor excess of chronic inflammatory cells within the submucosa. There was no evidence of bacteria, acid-fast bacilli or malignant cells.

Given the unusual airway appearances, further investigations were requested. The patient had a circulating ANCA against MPO titre (6.9 IU/ml, upper limit of normal 3.4 IU/ml). Complement levels were normal and there were no antibodies against anti-nuclear or anti-cyclic citrullinated peptides or extractable nuclear antigens. The patient was reviewed in the Vasculitis Clinic with ongoing right foot pain and tingling in both feet and weakness. Urinalysis now showed haematuria and mild proteinuria (urine protein:creatinine ratio 35 mg/mmol), however, kidney excretory function continued to be normal. A kidney biopsy showed a pauci-immune focal and necrotizing glomerulonephritis with cellular crescents and no scarring (Fig. 1D). He was treated with immunosuppression. His respiratory symptoms resolved rapidly and there was normalization of the systemic inflammatory response (Fig. 1E). Renal excretory function remained normal throughout. The neurological symptoms improved more gradually.

There are several pulmonary manifestations of systemic vasculitis, including interstitial lung disease, alveolar haemorrhage, tracheobronchial stenosis and pulmonary nodules (with or without cavitation). The pulmonary abnormality here was focussed on the airways, but there was no excess of tracheobronchial airway mucosal tissue and no evidence of stenosis. Interstitial lung disease may be part of a systemic autoimmune disease and a positive MPO ANCA is often found. However, many of these patients do not have other symptoms or signs consistent with a systemic vasculitis at initial diagnosis. Interestingly, a recent retrospective study demonstrated that MPO ANCA positivity in patients with idiopathic interstitial pneumonia is associated with future development of ANCA-associated vasculitis (AAV) [1]. Therefore it is important to comprehensively assess these patients at initial presentation and during follow-up for features of small vessel inflammation in other organs.

In this case, the combination of abnormal CT chest findings, positive ANCA serology, active urinary sediment and neurological symptoms was most in keeping with AAV affecting the airways and lung parenchyma, kidneys and nerves. This was confirmed by typical histology on kidney biopsy. The MPO ANCA was only modestly elevated; the ANCA titre correlates poorly with disease activity in AAV and having positive serology per se is more significant clinically than the titre itself. Conversely, in patients with a suggestive clinical presentation and/or compatible histology, the diagnosis can be made in the absence of positive ANCA testing [2]. In patients with active AAV, strawberry-like inflammation has been described in the gingiva [3]. This is the first time, to our knowledge, it has been described within the airways. Disease induction treatment here comprised two doses of intravenous cyclophosphamide and rituximab. This novel approach allowed a rapid glucocorticoid taper. A number of studies have investigated remission-induction regimens that allow a significant reduction in or complete avoidance of glucocorticoid exposure, often by using combinations of non-glucocorticoid immunosuppressive agents [4, 5].

Stonemasonry, particularly when working with stones such as granite ,which have a high quartz content, is known to increase the risk of developing silicosis. In this case, the lung parenchymal change was subtle, with the most striking abnormality being the presence of enlarged and calcified hilar and mediastinal lymph nodes. This highlights the role of the alveolar macrophage in clearing any silica deposited in the alveoli to the lymphatic system. The resulting inflammatory and fibrotic reaction results in enlarged glands that finally calcify. Once sufficient silica is inhaled to overwhelm and impair lung clearance, the same inflammatory and fibrotic processes occur in the parenchyma of the lung, leading to the development of silicosis. The typical changes of silicosis include bilateral upper lobe nodularity often accompanied by perilymphatic beading similar to that reported in cases of sarcoidosis, but so far as the authors are aware, the endobronchial changes described here have not been reported and we believe may represent a local reaction to inhaled silica.

In addition to silicosis, the inhalation of silica also increases the risk of developing pulmonary mycobacterial disease, lung cancer and chronic obstructive pulmonary disease and has been implicated in the pathogenesis of autoimmune conditions such as SSc, RA and SLE. This case highlights the potential role of respirable silica in the pathogenesis of AAV, which has been the subject of several case reports and case–control and cohort studies [6]. A recent study in France demonstrated a clear geospatial association between cases of granulomatosis with polyangiitis, a form of AAV, and the presence of quarries, a source of silica, which has been associated with the development of positive ANCA serology [7].

The evidence for the role of silica in the development of AAV is strongest in patients with glomerulonephritis and is underpinned by experimental evidence demonstrating how silica may contribute to the development of autoimmunity and chronic inflammation from which biologically plausible mechanisms may be postulated. However, while a few small studies have reported both an increased prevalence of ANCA and AAV in silica-exposed populations, the rarity of these conditions inevitably means that further large-scale studies with more clearly defined exposures are required before greater confidence in causation may be inferred. Nonetheless, the unresolved but potential role of silica in these conditions highlights the importance of adherence to strict occupational hygiene and the provision of personal protective equipment to all workers exposed to respirable silica.
